# Anti-Hyperpigmentation-Related Potential Activities in B16BL6 Cells and Chemical Composition of Essential Oil from *Chamaecyparis pisifera* Leaves

**DOI:** 10.3390/pharmaceutics17111386

**Published:** 2025-10-25

**Authors:** Do Yoon Kim, Kyung Jong Won, Yoon Yi Kim, Da Yeon Yoo, Hwan Myung Lee

**Affiliations:** 1 Department of Biotechnology, College of Bio-Health, Hoseo University, Asan 31499, Republic of Korea; doyoon@hoseo.edu (D.Y.K.); 20245588@vision.hoseo.edu (Y.Y.K.); 20245471@vision.hoseo.edu (D.Y.Y.); 2 Korea Essential Oil Resource Research Institute, Hoseo University, Asan 31499, Republic of Korea; 3 Department of Physiology and Premedical Science, College of Medicine, Konkuk University, Chungju 27478, Republic of Korea; kjwon@kku.ac.kr

**Keywords:** *Chamaecyparis pisifera*, essential oil, melanin, melanin synthesis, skin whitening, B16BL6 cells, tyrosinase, anti-hyperpigmentation

## Abstract

**Background/Objectives**: *Chamaecyparis pisifera* (*C. pisifera*; family Cupressaceae) is known to have insecticidal and antibacterial activities, but its effects on skin depigmentation-related activities have not been elucidated. Thus, in the present study, we aimed to investigate the anti-hyperpigmentation potential of *C. pisifera* var. filifera leaf essential oil (CPEO), specially focusing on responses related to melanogenesis and melanin transport, using B16BL6 cells. **Methods**: CPEO was extracted by steam distillation, and its composition was determined by GC/MS spectrometry. The biological activities of CPEO on B16BL6 melanoma cells were analyzed using the water soluble tetrazolium salt, BrdU incorporation, ELISA, and immunoblotting assays. **Results**: Twenty-eight components were identified in CPEO. CPEO was noncytotoxic to B16BL6 cells at 1–100 μg/mL and reduced serum-induced proliferation in B16BL6 cells. CPEO significantly inhibited α-MSH-stimulated increases in melanin synthesis and tyrosinase activity in the cells (e.g., at 100 μg/mL CPEO, melanin synthesis: 117.89 ± 0.00% vs. 571.94 ± 0.81% with α-MSH; tyrosinase activity: 73.62 ± 0.00% vs. 322.60 ± 3.10% with α-MSH). CPEO also downregulated the expression levels of melanogenesis-related proteins (MITF, tyrosinase, TRP-1 and -2) and melanosome transport-related proteins (Rab27a, melanophilin, myosin Va) in cells exposed to α-MSH. Moreover, the essential oil increased the phosphorylations of MAPKs (p38, ERK1/2, and JNK) in α-MSH-treated B16BL6 cells. In addition, CPEO reduced the ultraviolet A (UVA) induced increases in α-MSH levels in HaCaT cells. In addition, conditioned medium from HaCaT cells irradiated with UVA (CM-UVA) in the presence of CPEO reduced melanin synthesis and tyrosinase activity in B16BL6 cells (e.g., at CM-UVA with 100 μg/mL CPEO, melanin synthesis: 100.92 ± 0.99% vs. 134.44 ± 0.97% with CM-UVA; tyrosinase activity: 101.02 ± 1.81% vs. 133.77 ± 1.88% with CM-UVA). **Conclusions**: These findings suggest that CPEO inhibits melanin production (probably through the regulation of MAPKs) and transport-related activities in B16BL6 cells, and that CPEO may serve as a potential natural anti-hyperpigmentation or skin whitening.

## 1. Introduction

Melanin is a pigment synthesized by melanocytes, and these pigments are not just responsible for determining skin color but also provide protective functions, particularly against harmful ultraviolet (UV) radiation [1]. However, melanin overproduction and accumulation can lead to dermatological conditions such as solar lentigines, post-inflammatory hyperpigmentation, freckles, melasma, and melanoma [1,2,3], which can have a negative impact on the quality of life [3,4]. Thus, researchers are attempting to find ways to prevent or address hyperpigmentation and achieve more even skin tones [4].

Melanin biosynthesis is mediated by melanogenic enzymes and takes place in melanosomes located inside melanocytes [5]. These melanin-containing organelles move along dendrites toward the cell membrane and are finally transferred into surrounding keratinocytes. Thus, pigments are spread across the skin [5,6]. Therefore, any disruptions in the synthesis or transport of melanin can result in its overproduction and associated hyperpigmentation disorders [5,6].

Several factors influence melanin synthesis in melanocytes, such as UV exposure, certain medications, and hormonal signals like those generated by α-MSH (α-melanocyte-stimulating hormone) [1,2,7]. These factors promote melanogenesis, a process mediated by specific molecules, including the transcription factor MITF (microphthalmia-associated transcription factor) and enzymes such as tyrosinase, TRP-1 (tyrosinase-related protein-1), and TRP-2, in melanocytes [8]. Tyrosinase, the rate-limiting enzyme in this process, is responsible for converting the amino acid L-tyrosine into L-DOPA and later into L-DOPA quinone, which ultimately forms DOPA chrome [9]. TRP-1 and TRP-2 are important enzymes that contribute to the later steps of melanin production by processing intermediate compounds [9]. The enzyme TRP-1 is responsible for converting 5,6-dihydroxyindole-2-carboxylic acid (DHICA) to indole-5,6-quinone-2-carboxylic acid (IQCA) through an oxidation reaction, whereas TRP-2 facilitates the transformation of DOPA chrome into DHICA [9]. MITF acts as a central regulator of melanogenesis and is crucial for melanocyte viability and proliferation [8,10]. It also modulates transcription of melanogenic enzymes such as tyrosinase, TRP-1, and TRP-2 [11]. Importantly, MITF activity is modulated by the MAPK (mitogen-activated protein kinase) signaling pathway, which includes p38 MAPK, ERK (extracellular signal-regulated kinase), and JNK (c-Jun N-terminal kinase) subtypes, all of which contribute to the regulation of melanogenesis [10,12].

After melanin has been synthesized, it is transported within melanocytes to be distributed throughout the skin. This intracellular transport involves an actin-based mechanism that facilitates the movement of melanosomes toward the tips of melanocyte dendrites [5,8]. The movement of melanin-filled melanosomes within melanocytes is promoted by a protein complex composed of Rab27a, melanophilin (Slac2-a), and myosin Va (MyoVa) [5,8], and the formation of this protein complex is essential for actin-based movement of melanosomes [5]. Rab27a, a member of the small GTPase Rab family, mediates the location of melanophilin and myosin Va to the surface of the melanosome. This recruitment supports the anchoring of melanosomes to actin filaments, facilitating their movement toward the plasma membrane in melanocytes [13]. During this process, melanophilin acts as a key mediator by linking Rab27a to an actin-based motor protein, myosin Va, which moves melanosomes along the intracellular actin filaments [14,15].

The Cupressaceae family has traditionally been used in Korea, China, and Japan to alleviate neuralgia and as a known diuretic and aphrodisiac. Specifically, the genus Chamaecyparis continues to be used as a traditional medicine in these countries to treat these conditions [16,17]. Furthermore, the essential oil extracted from the Chamaecyparis genus has been shown to possess acaricidal, antibacterial, antifungal, antioxidant, antineoplastic, and insecticidal activities [18,19,20,21,22,23]. In particular, it has been reported that *C. pisifera* exhibits insecticidal activity [23], and that a methanol extract of its leaves has antibacterial effects [24]. *C. pisifera* var. filifera (also known as *C. pisifera* ‘Filifera’) is a variety of the species *C. pisifera* [25]. However, the effects of *C. pisifera* var. filifera on the biological activities of skin have not been examined. Thus, we investigated the effects of *C. pisifera* var. filifera leaf essential oil (CPEO) on skin depigmentation or whitening-related biological responses and related signals using melanocytes (B16BL6 melanoma cells).

## 2. Materials and Methods

### 2.1. Materials

Cell culture-related agents, including fetal bovine serum (FBS), trypsin-ethylenediamine tetra-acetic acid (EDTA), and penicillin/streptomycin (P/S) were supplied by Gibco BRL (Gaithersburg, MD, USA). Meanwhile, Dulbecco’s modified eagle medium (DMEM), Minimum essential medium (MEM), and phosphate-buffered saline (PBS) were provided by Welgene (Daegu, Republic of Korea). Additional reagents, such as L-DOPA, Triton X-100, bovine serum albumin (BSA), α-MSH, dimethyl sulfoxide (DMSO), and phenylmethylsulfonyl fluoride was procured from MilliporeSigma (St. Louis, MO, USA). EZ-CyTox kits were purchased from DoGenBio (Seoul, Republic of Korea), and cell proliferation enzyme-linked immunosorbent assay (ELISA) 5-bromo-2′-deoxyuridine (BrdU) kits were purchased from Roche (Mannheim, Germany). The antibodies used were sourced from several companies. Cell Signaling (Beverly, MA, USA) supplied anti-MITF, anti-Myosin Va, IgG antibodies (anti-mouse IgG and anti-rabbit IgG), and anti-MAPK antibodies (anti-JNK, p38 MAPK, anti-Erk1/2, anti- anti-phospho JNK, anti-phospho p38 MAPK, and anti-phospho Erk1/2). Abcam (Cambridge, UK) provided anti-TRP-1, anti-TRP-2, and anti-tyrosinase. The remaining antibodies used were anti-Rab27a (Santa Cruz Biotechnology, CA, USA); anti-melanophilin (Proteintech, Wuhan, China); anti-α-MSH (Bioss antibody Inc., Woburn, MA, USA); and β-actin (MilliporeSigma, St. Louis, MO, USA).

### 2.2. Preparation of C. pisifera var. filifera Leaf Essential Oil

*C. pisifera* var. filifera leaves were collected at the Department of Biotechnology, Hoseo University, Baebang-eup, Asan, Republic of Korea (34°44′14.00″ N 127°04′27.00″ E) on 3 August 2022, and identified by Hyun-Jun Kim at the Forest Medicinal Resources Research Center (Republic of Korea). For future reference, specimen No. 240408A1 was preserved at the Herbarium of the Korea Essential Oil Resource Research Institute and Korea Forest Plants Essential Oil Bank, Hoseo University. CPEO was derived from semi-dried *C. pisifera* var. filifera leaves via steam distillation. Its storage was held at 4 °C in light-protected vials. For experimental use, the CPEO was dispersed by dissolving it in PEG-40 hydrogenated castor oil (Sigma-Aldrich, St. Louis, MO, USA).

### 2.3. Analysis and Identification of Compounds in C. pisifera var. filifera Leaf Essential Oil

A gas chromatography/mass spectrometry (GC/MS) of CPEO was conducted at the National Instrumentation Center for Environmental Management (NICEM, Seoul, Republic of Korea) using an Agilent 7890BGC/7010QQQ MS instrument (Agilent Technologies, Santa Clara, CA, USA) equipped with a DB5-MS capillary column (30 m × 250 μm, film thickness 0.25 μm). Helium was used as the GC carrier gas at a flow rate of 1 mL/min. Injector port and interface temperatures were held at 300 °C, and the oven was programmed as follows: 40 °C for 2 min, 40 to 230 °C at 2 °C/min, 230 to 300 °C at 5 °C/min, and then held for 15 min at 300 °C. A split ratio of 1:10 was used. Mass spectra were acquired over an m/z range of 50 to 800, and retention indices (RIs) were calculated based on the Kovats method using standard C_7_-C_40_ n-alkanes. The identification of compounds was achieved by evaluating their retention indices against Kovats reference values [26], combined with comparison of their mass spectral fragmentation patterns to those available in the Wiley7NIST0.5L Mass Spectral databases. GC/MS data were re-analyzed as previously described [27].

### 2.4. Cell Culture

The murine melanoma cell line B16BL6 (sourced from the Intercellular Communication Network Lab, POSTECH, Pohang, Republic of Korea) and the human keratinocyte cell line HaCaT (obtained from the National Institute of Korean Medicine Development, NIKOM, Gyeongsan, Republic of Korea) were used in this study. All cells were grown in a humidified incubator at 37 °C with 5% CO_2_. B16BL6 cells were maintained in MEM, while HaCaT cells were cultured in DMEM; both media were supplemented with 1% P/S and 10% FBS. Experimental procedures were initiated when cells reached approximately 70–80% confluence.

### 2.5. Cell Viability Assay

A WST (water-soluble tetrazolium salt) assay was conducted to assess the viability of B16BL6 cells using the EZ-CyTox kit. Initially, 1.5 × 10^3^ cells were seeded per well into 96-well plates and allowed to attach. Subsequently, the adhered cells were exposed to varying concentrations of CPEO, which was solubilized in MEM containing 2% FBS and 0.02% PEG-40 hydrogenated castor oil. After the 48 h incubation, 10 μL of EZ-CyTox solution was pipetted into each well, and the plates were incubated at 37 °C for 30 min. The measurements of resulting colorimetric change were taken at 450 nm using a Synergy 2 multi-well plate reader (Bio-Tek Instruments, Winooski, VT, USA).

### 2.6. Proliferation Assay

Cell proliferation was assessed using an EZ-CyTox proliferation kit (DoGenBio, Seoul, Republic of Korea; a WST assay kit) and a BrdU incorporation assay kit (Roche, Indianapolis, IN, USA). For the EZ-CyTox kit proliferation assay, cells were seeded into 96-well cell culture plates at 1.5 × 10^3^ cells/well and treated with various concentrations of CPEO dissolved in MEM containing 0.02% PEG-40 hydrogenated castor oil and 2% FBS for 48 h in a humidified 95% air/5% CO_2_ atmosphere at 37 °C and then treated with EZ-CyTox reagent (10 μL/well) for 30 min under the same conditions. For the BrdU incorporation assay, 1.5 × 10^3^ B16BL6 cells were initially dispensed per well into 96-well plates and exposed to varying concentrations of CPEO dissolved in MEM containing 0.02% PEG-40 castor oil and 2% FBS for 36 h. BrdU-labeling solution (10 μM) was then added, and cells were incubated for 12 h at 37 °C to denature DNA. Cells were then treated with peroxidase-labeled anti-BrdU monoclonal antibody (Roche, Mannheim, Germany) and incubated for 90 min at room temperature (RT). BrdU antibody complexes were detected using a luminometer (Synergy 2, Bio-Tek Instruments, Winooski, VT, USA), and cell proliferation was assessed by expressing luminescence intensities as percentages of untreated controls.

### 2.7. Melanin Content Assay

Melanin content assay was performed according to previously detailed procedures [28,29]. B16BL6 cells were seeded at 5 × 10^4^ cells per well in 6-well plates and allowed to adhere for 12 h. After attachment, the cells were treated with diverse concentrations of CPEO in MEM (supplemented by 2% FBS), either in the presence or absence of 200 nM α-MSH, and incubated for 48 h at 37 °C. Following treatment, the cells were gently washed with PBS and lysed using a buffer solution containing 0.1 M sodium phosphate (pH 6.8; MilliporeSigma, St. Louis, MO, USA), 1% Triton X-100, and 0.2 mM phenylmethylsulfonyl fluoride (PMSF). Cell lysates were centrifuged at 10,000× *g* for 15 min, and the resulting pellets were collected. These pellets were thoroughly dissolved in 150 μL of 1 N NaOH containing 10% DMSO and incubated at 80 °C for 1 h to extract melanin. Absorbance was measured at 405 nm using a Synergy 2 ELISA microplate reader (Bio-Tek Instruments, Winooski, VT, USA) to determine melanin content.

### 2.8. Tyrosinase Activity Assays

The enzymatic activity of tyrosinase in B16BL6 cells was determined via dopachrome formation from L-DOPA, following previously established protocols [28,29]. Cells were cultured at 5 × 10^4^ cells per well in 6-well plates, lysed, and centrifuged, similarly to the melanin content assay. From the supernatants, 60 μL was transferred to a 96-well plate, mixed with 140 μL of 2 mg/mL L-DOPA, and incubated at 37 °C for 1 h. The absorbance at 490 nm was measured using a Synergy 2 ELISA reader (Bio-Tek Instruments, Winooski, VT, USA).

### 2.9. Western Blotting

To extract cellular proteins, cells were incubated with radioimmunoprecipitation assay (RIPA) buffer (Cell Signaling Technology, Beverly, MA, USA), and the resulting lysates were clarified by centrifugation at 17,000× *g* for 15 min at 4 °C. Protein concentrations in supernatants were determined using DC (detergent compatible) protein assay reagents (Bio-Rad Laboratories, Hercules, CA, USA). In addition, proteins (30–80 μg/lane) were separated by 10% SDS-PAGE and transferred to polyvinylidene fluoride membranes at 4 °C. These membranes were then incubated with 3% skim milk(BD difco, Sparks, MD, USA) or 3% BSA blocking solution at RT for 2 h, loaded with primary antibodies (1:1000–5000 dilution). Next, a subsequent incubation was performed with the horseradish peroxidase-coupled secondary antibody at RT for 60 min. Visualization of protein bands was achieved using a chemiluminescent reagent and then imaged with the LuminoGraph imaging system (ATTO, Tokyo, Japan).

### 2.10. UVA Irradiation

HaCaT cells (1 × 10^6^ cells per dish) were seeded in 100 mm dishes and cultured for 12 h. After washing with PBS, cells were exposed to 10 J/cm^2^ doses of UVA for different durations (38–40 min) using the BIO-LINK crosslinker (BLX; Vilber Lourmat, Collégien, France). The maximum wavelength (λmax) was maintained at 365 nm. For the Western blot assay, cells were incubated in serum-free MEM with or without CPEO at different concentrations for 24 h at 37 °C and lysed using RIPA buffer (Cell Signaling). The cell lysates were centrifuged (15 min, 4 °C), followed by a sequential Western blotting procedure, as described above. For the melanin content and tyrosinase activity assays, cells were exposed to UVA, as mentioned above. Cell media were then collected and underwent sequential centrifugation (500, 800, and 1500× *g*, with each step lasting for 10 min). The resulting supernatants (conditioned medium) were subsequently introduced to serum-free MEM with or without CPEO at different concentrations, added to 6-well plates seeded with B16BL6 cells (5 × 10^4^ cells/well) and cultured for 48 h at 37 °C. Melanin content and tyrosinase activity assays were performed as described above.

### 2.11. Statistical Analysis

Differences between pairs of groups were assessed for statistical significance using the Student’s t-test. Statistical comparison between multiple groups was executed using analysis of variance (ANOVA, one-way), followed by Tukey’s post hoc evaluation. These statistical calculations relied on GraphPad Prism (version 5.0; GraphPad Software, Inc., San Diego, CA, USA). Values are presented as means ± standard errors of means (SEMs), and *p* values of <0.05 were considered significant.

## 3. Results

### 3.1. Chemical Analysis of CPEO

**Figure 1:**
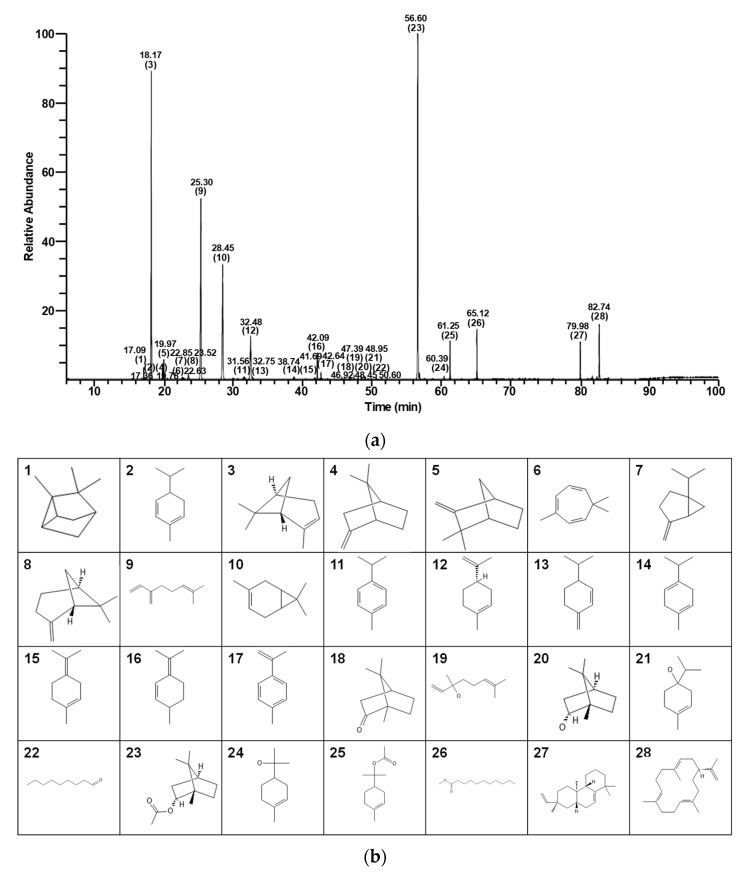
GC/MS total ion chromatogram of *C. pisifera* var. filifera leaf essential oil. (**a**) Bracketed numbers and numbers directly above them for each peak indicate numbers and retention times, respectively, of the 28 identified compounds (Table 1). (**b**) Chemical structures and compound numbers.

Analysis by GC/MS revealed the presence of 28 chemical compounds within CPEO sample (Figure 1 and Table 1). GC data showed that (-)-bornyl acetate was most abundant (26.72%), followed by (+)-α-pinene (21.33%), myrcene (17.51%), 3-carene (12.47%), (-)-limonene (5.04%), neocembrene (3.26%), methyl undecanoate (2.17%), terpinyl acetate (2.01%), and other compounds (Table 1).

### 3.2. Effects of CPEO on the Viability and Proliferation of B16BL6 Cells

**Figure 2:**
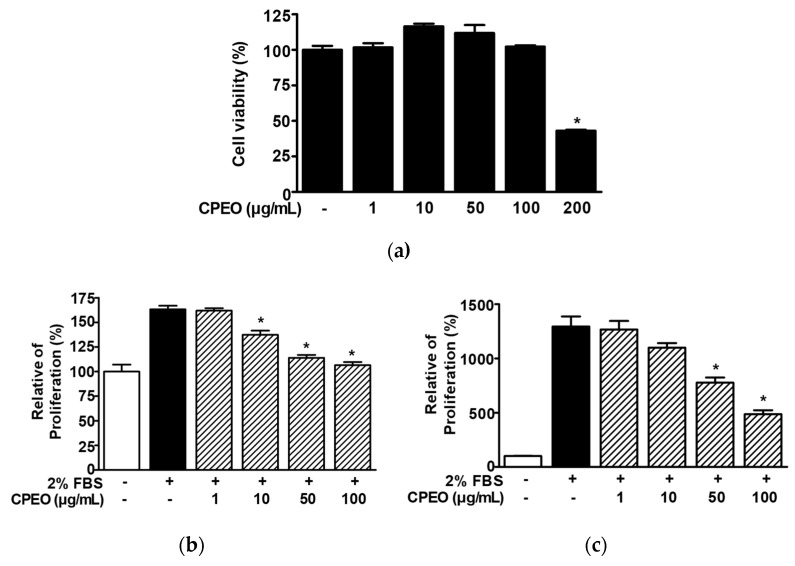
Effects of *C. pisifera* var. filifera leaf essential oil on B16BL6 cell viability and proliferation. (**a**) Cell viability was assessed after 48 h of incubation with varying concentrations of *C. pisifera* var. filifera leaf essential oil (CPEO; 1–200 μg/mL), using the WST assay (*n* = 3). (**b**,**c**) To assess proliferation analysis, cells were maintained in MEM supplemented or not with 2% FBS, in the presence or absence of CPEO (1–100 μg/mL) for 48 h, and proliferation was subsequently evaluated using the WST assay ((**b**); *n* = 3) and the BrdU incorporation assay ((**c**); *n* = 3) as described in Materials and Methods. The cell viability (**a**) and proliferation (**b**,**c**) levels of the untreated controls (-) were defined to be 100%. Data are expressed as mean ± SEMs. * *p* < 0.05 vs. untreated controls (**a**) or 2% FBS alone-treated cells (**b**,**c**).

In order to evaluate how CPEO influences melanogenesis, we first examined the toxic effect of CPEO (1–200 μg/mL) on B16BL6 melanoma cells using the WST assay. CPEO did not significantly affect B16BL6 melanoma cell viability at concentrations of 1 to 100 μg/mL but significantly reduced B16BL6 melanoma cell viability at 200 μg/mL (Figure 2a). Next, we examined whether CPEO (1–100 μg/mL) affected the proliferation of B16BL6 cells using WST and BrdU assays. CPEO exhibited an inhibitory effect on B16BL6 cell proliferation stimulated by 2% FBS, showing significant suppression at 10–100 μg/mL in the WST assay and at 50 and 100 μg/mL in the BrdU assay. Maximal effects as determined by proliferation assays were observed at 100 μg/mL (106.55 ± 3.12% and 486.50 ± 36.05% of untreated control, Figure 2b and Figure 2c, respectively).

### 3.3. Changes in Melanin Synthesis and Tyrosinase Activity in CPEO-Exposed B16BL6 Cells

**Figure 3:**
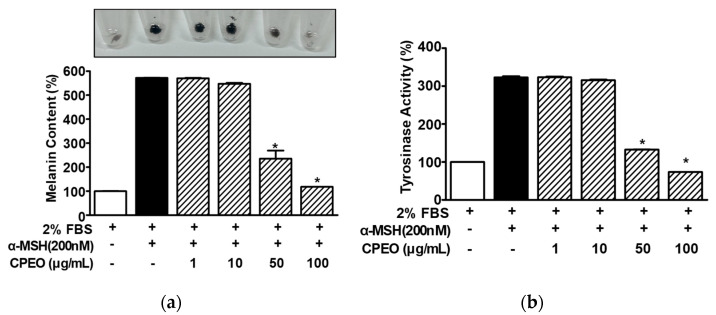
Effects of *C. pisifera* var. filifera leaf essential oil on α-MSH-induced melanin synthesis and tyrosinase activity in B16BL6 cells. Cells were cultured for 48 h with or without *C. pisifera* var. filifera leaf essential oil (CPEO; 1–100 μg/mL; diluted with MEM containing 2% FBS) in the presence or absence of α-melanocyte-stimulating hormone (α-MSH: 200 nM). Melanin content ((**a**); *n* = 3) and tyrosinase activity ((**b**); *n* = 3) were assayed following the procedures detailed in Section 2. A representative image is displayed in the upper part of panel (**a**). α-MSH was used as a positive control. FBS (2%) control values were defined as 100%. Data are presented as means ± SEMs. * *p* < 0.05 vs. the α-MSH alone-treated cells in the presence of FBS (2%).

To assess the impact of CPEO on melanin synthesis, B16BL6 melanoma cells were stimulated with α-MSH (200 nM) with or without CPEO (1–100 μg/mL) and subjected to melanin content analysis. α-MSH (200 nM) elevated melanin levels in B16BL6 cells (by 571.94 ± 0.81% versus the FBS (2%) alone-treated control), and this increase was significantly reduced by CPEO (50 and 100 μg/mL). The maximum effect was observed at a CPEO concentration of 100 μg/mL (117.89 ± 0.00% versus the FBS (2%) alone-treated control; Figure 3a). We also examined the effect of CPEO on tyrosinase activity. CPEO (50 and 100 μg/mL) significantly reduced α-MSH (200 nM)-increased tyrosinase activity in B16BL6 cells (by 322.60 ± 3.10% versus the FBS (2%) alone-treated control). The maximal effect was observed at 100 μg/mL (73.62 ± 0.00% versus the FBS (2%) alone-treated control; Figure 3b).

### 3.4. Changes in the Expressions of Melanogenesis-Regulator Molecules in CPEO-Exposed B16BL6 Cells

**Figure 4:**
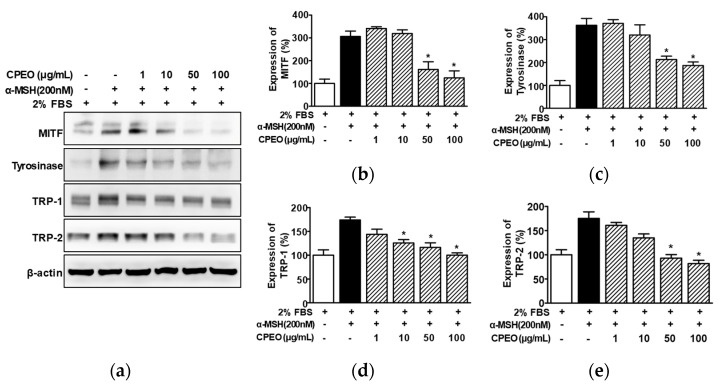
Effect of *C. pisifera* var. filifera leaf essential oil on the levels of proteins related to melanogenesis in B16BL6 cells. (**a**) Typical images of the findings. The cells were incubated in culture media with or without CPEO (*C. pisifera* var. filifera leaf essential oil) at 1–100 μg/mL (diluted with MEM containing 2% FBS) in the presence or absence of α-melanocyte-stimulating hormone (α-MSH: 200 nM) for 24 h. According to the procedures detailed in Section 2, immunoblotting was carried out on cell lysates using specific antibodies. (**b**–**e**) Statistical quantification (*n* = 3) of melanogenic protein levels, including microphthalmia-associated transcription factor (MITF) (**b**), (*n* = 3), tyrosinase (**c**), tyrosinase-related protein-1 (TRP-1) (**d**), and tyrosinase-related protein-2 (TRP-2) (**e**). α-MSH: the positive control. Expressions in 2% FBS alone-treated cells were defined as 100%. All values are reported as means ± SEMs; the asterisk (*) indicates a significant difference (*p* < 0.05) compared to cells treated with α-MSH alone under 2% FBS conditions.

To elucidate how melanogenesis and tyrosinase activity levels are inhibited in B16BL6 melanoma cells exposed to CPEO, we conducted Western blotting. We specially focused on the actions of CPEO on key melanogenic enzymes (tyrosinase, TRP-1, and TRP-2) and the regulating transcription factor, MITF. When B16BL6 cells were stimulated with α-MSH (200 nM), the expression level of MITF rose substantially, reaching 310.32 ± 17.70% relative to the control group treated with 2% FBS alone (Figure 4a,b). This increase was significantly decreased in B16BL6 cells by CPEO (50 and 100 μg/mL), and the maximum effect was observed at 100 μg/mL of CPEO (to an increase of 126.25 ± 24.19% versus the FBS (2%) alone-treated control; Figure 4a,b). Furthermore, the increase in the level of tyrosinase in B16BL6 cells induced by 200 nM α-MSH (367.38 ± 24.62% versus the FBS (2%) alone-treated control) was significantly decreased by CPEO at 50 and 100 μg/mL and this inhibition was maximum at 100 μg/mL (to an increase of 188.92 ± 10.46% versus the FBS (2%) alone-treated control; Figure 4a,c). CPEO also significantly reduced α-MSH-mediated increases in TRP-1 within B16BL6 cells across a concentration range of 10 to 100 μg/mL. This suppressive action was most pronounced at 100 μg/mL, where the TRP-1 was attenuated from 176.52 ± 3.05% to 101.52 ± 2.13% compared to the FBS (2%) alone-treated control group (Figure 4a,d). Furthermore, CPEO significantly diminished the α-MSH-elevated expression of TRP-2 in B16BL6 cells. This significant inhibitory effect was observed starting at 50 μg/mL and maximized at 100 μg/mL, achieving a reduction to 101.52 ± 2.13% from 177.68 ± 11.01% relative to the FBS (2%) alone-treated control (Figure 4a,e).

### 3.5. Altered Levels of Melanosome Transport-Associated Proteins in B16BL6 Cells Following CPEO Treatment

**Figure 5:**
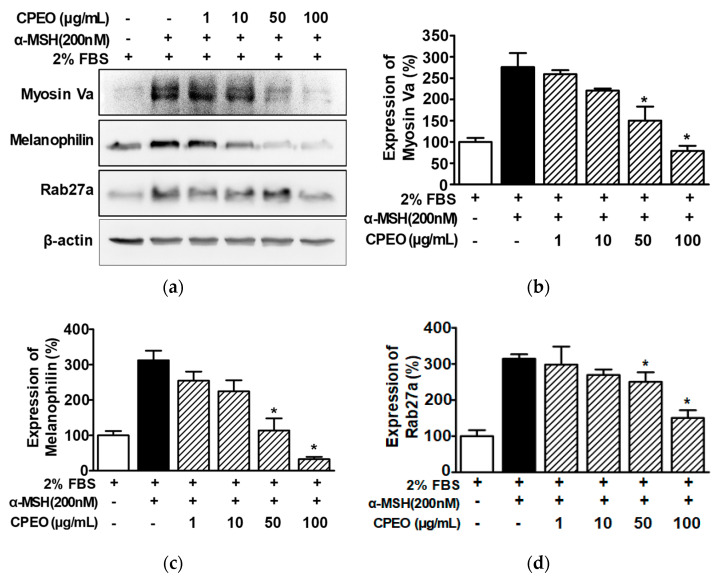
Effect of *C. pisifera* var. filifera leaf essential oil on the levels of proteins related to melanosome transport in B16BL6 cells. (**a**) Typical images of the findings. The cells were incubated in culture media with or without CPEO (*C. pisifera* var. filifera leaf essential oil) at 1–100 μg/mL (diluted with MEM containing 2% FBS) in either with or without 200 M α-MSH (α-melanocyte-stimulating hormone) for 24 h. According to the procedures detailed in Section 2, immunoblotting was carried out on cell lysates each test antibody. (**b**–**d**) Statistical graphs illustrating myosin Va (**b**), melanophillin (**c**), and Rab27a expression levels (**d**) derived from panel (**a**). α-MSH was used as the positive control. Protein expression levels are presented as the relative change compared to cells treated with 2% FBS alone. Data are reported as means ± SEMs, with three independent experiments (*n* = 3) per protein. * *p* < 0.05 vs. α-MSH alone-treated cells in the presence of FBS (2%).

To investigate the effect of CPEO on melanosome transport, we evaluated the effect of CPEO on the expression of melanosome transport proteins (myosin Va, melanophilin, and Rab27a) by performing Western blotting on B16BL6 cells exposed to α-MSH under 2% FBS. α-MSH (200 nM) increased the expression of myosin Va (by 279.73 ± 28.66% of the 2% FBS alone-treated control; Figure 5a,b). This was significantly inhibited in B16BL6 cells at 50 and 100 μg/mL of CPEO, with maximal effect at 100 μg/mL (by 79.82 ± 7.50% of the 2% FBS alone-treated control; Figure 5a,b). Additionally, CPEO significantly reduced the α-MSH (200 nM)-induced expressions of melanophilin (Figure 5a,c) and Rab27a (Figure 5a,d) in B16BL6 cells at 50 and 100 μg/mL. The lowest expression for these proteins occurred at 100 μg/mL of CPEO, with melanophilin at 32.75 ± 1.07% (Figure 5c) and Rab27a at 152.12 ± 16.99% (Figure 5d) relative to the 2% FBS alone-treated control.

### 3.6. Changes in the Activations of MAPKs in CPEO-Treated B16BL6 Cells

**Figure 6:**
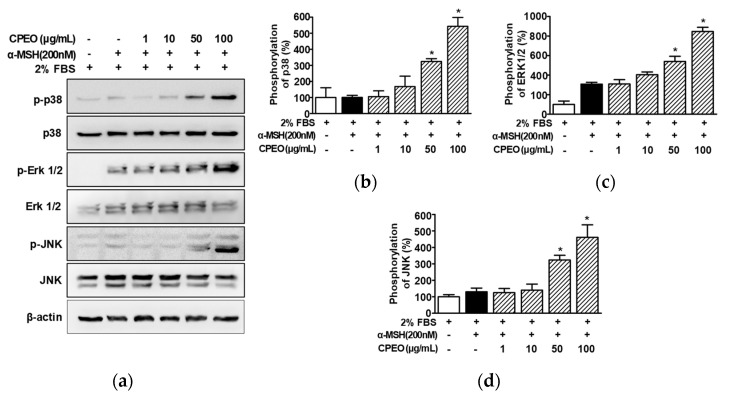
Effect of *C. pisifera* var. filifera leaf essential oil on the activation of kinases in B16BL6 cells. (**a**) Representative image results. *C. pisifera* var. filifera leaf essential oil (CPEO; 1–100 μg/mL; diluted with MEM containing 2% FBS) with or without 200 nM α-melanocyte-stimulating hormone (α-MSH) for 5 min. According to the procedures detailed in Section 2, immunoblotting was carried out on cell lysates (**b**–**d**). Statistical graphs of the expression levels of phosphorylated p38 MAPK (p-p38: (**b**)), ERK1/2 (p-ERK 1/2: (**c**)), and JNK (p-JNK: (**d**)) derived from panel (**a**). Positive control: α-MSH. Protein expression levels are presented as the relative change compared to cells treated with 2% FBS alone. All values are reported as means ± SEMs; the asterisk (*) indicates a significant difference (*p* < 0.05) compared to cells treated with α-MSH alone under 2% FBS conditions.

MAPKs are regulatory signaling molecules involved in melanogenesis and MITF [10,12]. To determine whether CPEO affects the upstream signaling molecules of melanogenesis regulation, we assessed the impact of CPEO (1–100 μg/mL) on the activation of p38 MAPK, ERK1/2, and JNK in B16BL6 melanoma cells treated with 200 nM α-MSH under 2% FBS conditions, using Western blot analysis. α-MSH (200 nM) induced p38 MAPK phosphorylation (by 101.83 ± 4.59% versus the 2% FBS control; Figure 6a,b) in B16BL6 cells, and this was significantly elevated in the presence of CPEO at 50 and 100 μg/mL, with maximal effect at 100 μg/mL (by 551.38 ± 47.71% versus the 2% FBS control; Figure 6a,b). In addition, α-MSH (200 nM) induced ERK1/2 (by 311.07 ± 5.73% versus the 2% FBS control; Figure 6a,c) and JNK phosphorylations (by 131.43 ± 13.71% versus the 2% FBS control; Figure 6a,d), and CPEO at 100 μg/mL maximally increased these phosphorylations (by 858.78 ± 28.63% [Figure 6c] and 467.18 ± 69.90% [Figure 6d], respectively).

### 3.7. Effect of CPEO on Tyrosinase Activity and Melanin Synthesis in B16BL6 Cells Exposed to Conditioned Medium Collected from UVA-Irradiated HaCaT Cells

**Figure 7:**
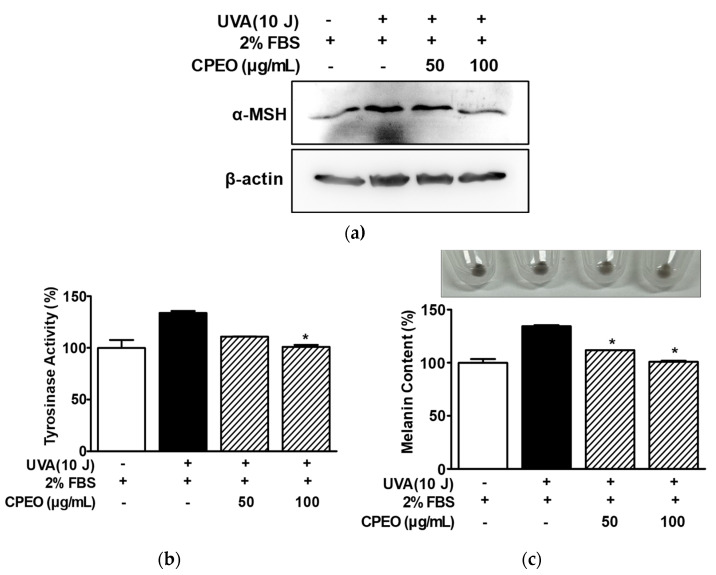
Effects of *C. pisifera* var. filifera leaf essential oil in the presence of conditioned medium of UVA-irradiated keratinocytes on tyrosinase activity and melanin synthesis in B16BL6 cells. (**a**) UVA-induced α-MSH expression in HaCaT cells. Cells were irradiated or not with 10 J/cm^2^ UVA for 38–40 min and then incubated with or without *C. pisifera* var. filifera leaf essential oil (CPEO; 50 or 100 μg/mL; diluted with MEM containing 2% FBS) for 24 h. Immunoblot analysis of cell lysates was conducted using the specified antibodies (*n* = 3). (**b**,**c**) Tyrosinase activity and melanin contents in B16BL6 cells. HaCaT cells were exposed or not exposed to 10 J/cm^2^ of UVA for 38–40 min. Conditioned media were collected by centrifugation as described in Materials and Methods. B16BL6 cells were incubated in conditioned media with or without *C. pisifera* var. filifera leaf essential oil (CPEO; 50 or 100 μg/mL diluted with MEM containing 2% FBS) for 48 h, and then tyrosinase activity ((**b**); n = 3) and melanin content ((**c**); *n* = 3) were assessed according to the procedures detailed in Section 2. Representative image is shown in the upper section of panel (**c**). Conditioned medium of HaCaT cells irradiated with 10 J/cm^2^ UVA was used as a positive control. FBS (2%) treated control values were defined as 100%. Data are presented as means ± SEMs. * *p* < 0.05 vs. the conditioned medium of UVA (10 J/cm^2^) irradiated HaCaT cells in the presence of FBS (2%).

Keratinocytes in skin exposed to UVA produce α-MSH, which increases melanin synthesis-related molecules and melanin levels in melanocytes [30,31]. Thus, we investigated the effect of CPEO on melanin production in melanocytes treated with the conditioned medium of HaCaT cells exposed to UVA. Initially, Western blotting showed that UVA (10 J/cm^2^) increased α-MSH production in HaCaT cells (in the presence of 2% FBS) and that this was markedly reduced by CPEO at 100 μg/mL (Figure 7a). Next, to determine whether CPEO affects tyrosinase activity and melanin synthesis in B16BL6 melanoma cells exposed to α-MSH induced in UVA-irradiated HaCaT cells, we collected conditioned medium from UVA (10 J/cm^2^)-treated HaCaT cells. We then evaluated the effect of CPEO (50 or 100 μg/mL) on tyrosinase activity and melanin synthesis in B16BL6 cells exposed to conditioned medium in the presence of 2% FBS. B16BL6 cells exposed to CPEO free-conditioned medium of HaCaT cells irradiated with UVA showed increased tyrosinase activity (by 133.77 ± 1.88% of the FBS (2%) control), and this increase was significantly reduced by CPEO (100 μg/mL)-containing conditioned medium of UVA-treated HaCaT cells (to 101.02 ± 1.81% of the FBS (2%) control; Figure 7b). Additionally, the CPEO free-conditioned medium of HaCaT cells irradiated with UVA significantly increased melanin synthesis in B16BL6 cells (by 134.44 ± 0.97% versus the FBS (2%) control) (Figure 7c), and this increase in melanin synthesis was significantly reduced by CPEO (50 or 100 μg/mL)-containing the conditioned medium of UVA irradiated HaCaT cells (Figure 7c). This response peaked when B16BL6 cells were treated with 100 μg/mL CPEO-containing conditioned medium of HaCaT cells irradiated with UVA (by 100.92 ± 0.99% of FBS (2%) alone-treated control; Figure 7c).

## 4. Discussion

In the present study, we found that CPEO not only inhibited UVA-induced α-MSH in keratinocytes but also downregulated melanogenesis-related molecules (MITF, tyrosinase, TRP-1, TRP-2) and melanin production in B16BL6 melanoma cells. Furthermore, CPEO modulated the expression of MAPKs and melanosome transport proteins. Thus, this study demonstrates the potential of CPEO to promote skin depigmentation or whitening-related responses in B16BL6 melanoma cells. The majority of plant-based skin whitening-related agent studies primarily focused on simple enzyme inhibition (e.g., tyrosinase inhibition) and antioxidant effects, with limited exploration of deeper signaling pathways (e.g., MAPK and CREB/AKT) or other biological effects [32]. Therefore, our study indicates that CPEO is a novel natural substance with application potential that differentiates it from existing herbal skin depigmentation and whitening agents. Many skin depigmentation and whitening agents have limited efficacy and exhibit cytotoxicity, which prompts searches for safer, more effective alternatives. Natural products, especially plant extracts, are considered a promising source of such alternatives because of their low toxicities and high bioactivities [2,33,34]. *C. pisifera* is known to possess insecticidal and antibacterial effects [23,24], but the effects of this plant and a variant *C. pisifera* var. filifera on skin biological activity, especially skin depigmentation or whitening-related activities, have not been studied. Thus, we extracted CPEO from *C. pisifera* var. filifera and observed whether CPEO affected melanogenesis-related activities in B16BL6 melanoma cells. We identified twenty-eight compounds in CPEO by GC/MS. The effects of these compounds on melanogenesis-related responses in B16BL6 melanoma cells were not directly tested in the present study. This topic will be addressed by future experiments designed to identify their skin whitening-related anti-melanogenesis effects. However, among the identified compounds, some compounds, such α-pinene, sabinene, and myrcene, were reported to inhibit melanin production through downregulation of several melanogenesis-linked responses [35,36,37]. Therefore, CPEO may have the potential to promote skin depigment activities by inhibiting melanogenesis. B16BL6 cell line, derived from a mouse melanoma, is widely used as a stable in vitro model for assessing anti-hyperpigmentation or skin-whitening effects because of its consistency regarding melanin synthesis [29]. CPEO did not significantly affect B16BL6 cell viability at concentrations of 1–100 μg/mL, indicating that at these concentrations, it is not toxic to B16BL6 melanoma cells. Thus, CPEO in the concentration range 1–100 μg/mL was used to determine its effects on B16BL6 cells.

Melanin production in melanocytes is regulated by factors that influence melanocyte survival, proliferation, and differentiation [38]. A decrease in melanocyte proliferation suppresses melanin production [39]; we found that CPEO attenuated serum-induced cell proliferation in B16BL6 cells. Moreover, CPEO reduced α-MSH-induced melanin content level in B16BL6 cells incubated in MEM containing 2% FBS, indicating CPEO inhibits melanin production in melanocytes by suppressing proliferation.

Melanin overproduction results in skin hyperpigmentation and can be prevented by inhibiting melanogenesis [40]. Furthermore, it has been reported that MITF, a crucial transcription factor, promotes melanin synthesis by inducing tyrosinase and tyrosinase-related proteins (TRP-1 and TRP-2) [9]. Conversely, a reduction in MITF has been shown to lower the expression of these melanogenesis-related proteins and decrease melanin content within B16F10 cells [41]. The findings from these reports highlight the role of MITF as a principal upstream regulator in the melanocyte melanin synthesis cascade. The current study similarly observed that CPEO mitigated α-MSH-enhanced MITF expression and downregulated tyrosinase, TRP-1, and TRP-2 in B16F10 cells. Tyrosinase, TRP-1, and TRP-2 are crucial mediators of melanogenesis [2]. Tyrosinase is the rate-limiting enzyme of melanogenesis and plays a pivotal role in the first two steps of melanin formation, namely, the hydroxylation of L-tyrosine to L-DOPA, and the subsequent oxidation of L-DOPA to L-DOPA quinone [9]. In addition, melanin production is intimately connected to the activity and protein level of tyrosinase [42], and it has been demonstrated that reducing tyrosinase activity and expression, or introducing a tyrosinase inhibitor, effectively suppresses melanin levels in B16BL6 cells exposed to α-MSH [43]. Similarly, in the current study, we observed that treatment of B16BL6 cells with CPEO lowered α-MSH-induced tyrosinase activity and expression levels. Moreover, we found that CPEO attenuated the upregulation of TRP-1 and TRP-2 as well as melanin production triggered by α-MSH stimulation in B16BL6 cells. Consistently, Joo et al. [43] showed that the suppression of melanin production in α-MSH-treated B16F10 cells is promoted by lowering TRP-1 and TRP-2 levels. Therefore, our results suggest that CPEO reduces melanin synthesis due to the inhibition of MITF expression and subsequent reductions in the levels of key melanogenic enzymes such as tyrosinase, TRP-1, and TRP-2.

Melanin is synthesized in melanocyte melanosomes, which are transported to the tips of cellular dendrites by actin-based motor proteins. Subsequently, melanosomes are transferred to keratinocytes via exocytosis, which results in the distribution of melanin in skin [6,44]. This process suggests that melanosome transport plays a crucial role in skin pigmentation. Actin-mediated melanosome transport in melanocytes relies on a critical three-protein complex, comprising Rab27a, melanophilin, and myosin Va [45,46,47,48]. When any protein within this tripartite is deficient, defective, or impaired, melanin transport is disrupted, which leads to abnormal melanosome distribution and hypopigmentation, which is often observed as the peri-nuclear aggregation of melanosomes [5,47,49]. These findings imply that modulating these key melanosome transport molecules might provide a viable strategy for achieving skin whitening and depigmentation. Previous studies have demonstrated the crucial roles of specific proteins in melanosome transport. For instance, reducing Rab27a activity diminished both the formation of melanin and its movement within melanocytes exposed to UVB radiation [46]. In another study, interfering with myosin Va isoforms halted melanosome transport entirely within these cells [50]. Additionally, lower levels of melanophilin and myosin Va were found to inhibit melanosome transport and reduce pigmentation [51]. Similarly, a decrease in Rab27a, melanophilin, and myosin Va expression has been shown to suppress melanin transport and skin pigmentation in human skin models and in vivo, suggesting these molecules are promising targets for regulating pigmentation [52]. In the present study, CPEO decreased α-MSH-elevated levels of melanophilin, Rab27a, and myosin Va protein in B16BL6 cells. Therefore, CPEO seems capable of inhibiting melanosome transport by reducing the expressions of myosin Va, melanophilin, and Rab27a, and of inhibiting melanogenesis within melanocytes. Taken together, this study suggests that CPEO has a dual effect by simultaneously impeding intracellular melanosome transport and melanin production, which could be helpful for lightening or brightening skin.

ERK1/2, p38 MAPK, and JNK are members of the MAPK family and have significant roles in signaling pathways involved in melanogenesis. They are also essential upstream regulators of MITF, which in turn controls the expression of tyrosinase, TRP-1, and TRP-2 [9,12]. Studies have shown that the downregulation of the phosphorylation of p38 MAPK, JNK, and ERK1/2 can reduce MITF levels and subsequently inhibit melanin production in B16F10 cells [9,44,53]. Conversely, it has been reported that a decrease in melanin content level is associated with the inhibition of melanogenic proteins, such as tyrosinase level and activity and MITF expression via the elevation of p38 MAPK, JNK, and ERK phosphorylation in melanocytes [54,55]. Furthermore, reductions in p38 MAPK level, JNK phosphorylation, and increases in ERK1/2 level inhibited melanin production by reducing MITF level in B16F10 cells [9,56]. Other findings indicate that the upregulations of p38 MAPK and ERK1/2 phosphorylation also downregulate MITF and tyrosinase, thereby inhibiting melanogenesis [57]. Also, it was reported that an increase in ERK1/2 activation without changes in the activations of p38 and JNK reduced levels of melanogenic proteins, including MITF, and led to a reduction in melanin production [58]. These reports suggest that melanin production can be suppressed by inhibiting MITF in melanocytes by decreasing or increasing MAPK phosphorylation, or through changes in MAPK phosphorylation. In the current study, we observed that CPEO suppressed MITF expression and enhanced the activation of JNK, ERK1/2, and p38 MAPK through phosphorylation following α-MSH exposure in B16BL6 cells. Moreover, CPEO reduced melanin content and lowered the levels of major melanogenesis-related proteins, namely tyrosinase, TRP-1, and TRP-2 in these cells. Given that MITF is a master regulator that stimulates the expression of melanogenic proteins to promote melanin synthesis [9], our findings indicate that CPEO suppresses melanogenesis, potentially by reducing MITF level, which is likely mediated by the MAPK signaling pathway.

To determine whether CPEO inhibits UV-derived melanogenesis in skin, we tested CPEO effects on melanin production in melanocytes using the conditioned medium collected from UV-irradiated keratinocytes. We found that UVA-induced increases in α-MSH levels were downregulated in CPEO-treated HaCaT cells, which implied that CPEO has an anti-melanogenic effect in UVA-irradiated keratinocytes. Moreover, CPEO reduced melanin production and tyrosinase activity in B16BL6 melanocytes in the presence of conditioned medium collected from HaCaT cells irradiated with UVA. Previous studies showed that UVA-irradiation induced α-MSH in HaCaT cells and that conditioned medium collected from UVA-irradiated HaCaT cells in the presence of 3-O-ethyl ascorbic acid or ectoine inhibited the expressions of melanogenic proteins and melanin and tyrosinase activity levels in B16F10 melanoma cells [30,31,59]. The inhibitory effect of 3-O-ethyl ascorbic acid or ectoine on melanin synthesis in α-MSH-exposed B16F10 cells were associated with a decrease in tyrosinase and TRP-1/-2 expression via inhibition of MITF expression [31,59]. In addition, we showed that CPEO treatment markedly suppressed the levels of both melanin and melanogenesis-related proteins in B16F10 cells following α-MSH exposure. Our findings indicate that CPEO exhibits anti-melanogenic activity against UVA-irradiated melanogenesis, suggesting a complex functional relationship between keratinocytes and melanocytes in the melanogenesis pathway.

In summary, this study identified 28 compounds in CPEO. Our findings reveal that CPEO suppresses the serum-stimulated proliferation of B16BL6 melanoma cells and inhibits both melanin biosynthesis and tyrosinase enzymatic activity in response to α-MSH stimulation. Moreover, treatment with CPEO led to a decrease in the expression of melanogenesis-related factors, namely tyrosinase, TRP-1, TRP-2, and MITF in B16BL6 cells stimulated with α-MSH. It was also observed that CPEO attenuated the activation of critical signaling molecules, such as ERK1/2, p38 MAPK, and JNK. The levels of melanosome transport proteins, especially myosin Va, melanophilin, and Rab27a, were decreased by CPEO under α-MSH-stimulated conditions. Furthermore, UVA exposure was found to elevate α-MSH levels in HaCaT keratinocytes, and this effect was diminished in the presence of CPEO. When B16BL6 cells were cultured in conditioned media derived from UVA-irradiated HaCaT cells treated with CPEO, melanin synthesis and tyrosinase activity were markedly reduced. Taken together, these results suggest that CPEO may exert a depigmenting effect by inhibiting both melanogenic pathways and melanosome transport mechanisms in melanocytes, making it a natural candidate for developing anti-hyperpigmentation and skin whitening agents. Future investigations are warranted to isolate and identify the active compounds within CPEO responsible for its inhibitory effects. Additionally, the action of CPEO in a practical skin application requires further investigation into skin penetration and uptake.

## Figures and Tables

**Table 1:** Components in essential oil extracted from *C. pisifera* var. filifera leaves.

No	Component Name	RT ^1^ (min)	RI ^2^		Area (%)
			Observed	Literature	
1	Tricyclene	17.09	918	918	0.78
2	α-Phellandrene	17.36	920	920	0.12
3	(+)-α-Pinene	18.17	927	929	21.33
4	α-Fenchene	19.78	941	941	0.69
5	Camphene	19.97	942	942	1.52
6	3,7,7-trimethylcyclohepta-1,3,5-triene	22.63	964	970	0.18
7	Sabinene	22.85	966	966	0.09
8	β-Pinene	23.52	972	972	0.50
9	Myrcene	25.30	987	987	17.51
10	3-Carene	28.45	1009	1009	12.47
11	P-Cymene	31.56	1028	1028	0.35
12	(-)-Limonene	32.48	1033	1031	5.04
13	β-Phellandrene	32.75	1035	1035	0.11
14	γ-Terpinene	38.74	1071	1071	0.25
15	Terpinolene	41.69	1089	1089	0.11
16	Isoterpinolene	42.09	1091	1091	1.67
17	α,p-Dimethylstyrene	42.64	1094	1094	0.45
18	Camphor	46.92	1151	1151	0.09
19	Linalool	47.39	1158	1140	0.19
20	(-)-Borneol	48.45	1175	1173	0.17
21	4-Carvomenthenol	48.95	1182	1182	0.12
22	Nonanal	50.60	1206	1206	0.05
23	(-)-Bornyl acetate	56.60	1285	1285	26.72
24	α-Terpineol	60.39	1342	1231	0.17
25	Terpinyl acetate	61.25	1356	1356	2.01
26	Methyl undecanoate	65.12	1415	1414	2.17
27	Rimuene	79.98	1911	1914	1.86
28	Neocembrene	82.74	1948	1941	3.26
Total Identified (%)					100.00

^1^ RT: Retention time, ^2^ RT: Retention indices determined using a DB-5MS capillary column.

## Data Availability

The datasets used and/or analyzed during the current study are available from the corresponding author upon request..

## Abbreviations

The following abbreviations are used in this manuscript:

α-MSH	α-Melanocyte-stimulating hormone
BSA	Bovine serum albumin
BrdU	5-Bromo-2′-deoxyuridine
CPEO	*C. pisifera* var. filifera leaf essential oil
DMEM	Dulbecco’s modified eagle medium
FBS	Fetal bovine serum
GC/MS	Gas chromatography/mass spectrometry
MEM	Minimum essential medium
P/S	Penicillin/streptomycin
RT	Room temperature
UV	Ultraviolet
